# Human brain matters: Navigating the neuropathology of COVID‐19

**DOI:** 10.1111/bpa.70101

**Published:** 2026-04-22

**Authors:** Juliana M. Nieuwland, Angelica Scaramuzza, Marianna Bugiani, Wilma D. J. van de Berg, Jinte Middeldorp

**Affiliations:** ^1^ Department of Neurobiology and Aging Biomedical Primate Research Centre Rijswijk The Netherlands; ^2^ Department of Pathology, Amsterdam UMC Vrije University Amsterdam Amsterdam The Netherlands; ^3^ Section Clinical Neuroanatomy and Biobanking and Life Sciences, Department of Anatomy and Neurosciences, Amsterdam UMC Vrije University Amsterdam Amsterdam The Netherlands; ^4^ Neurodegeneration, Amsterdam Neuroscience Amsterdam The Netherlands

**Keywords:** long COVID, neuropathology, PASC, protein aggregation, SARS‐CoV‐2, severe COVID‐19, vasculature

## Abstract

Infection with severe acute respiratory syndrome coronavirus 2 (SARS‐CoV‐2) has caused millions of deaths worldwide. Although the incidence of severe acute cases has declined, the prevalence of long COVID, also known as post‐acute sequelae of COVID‐19 (PASC), is rising. The pathological mechanisms underlying severe COVID‐19, along with the relationship to neurological disorders and potential risk for neurodegeneration, remain poorly understood. The aim of this narrative review is to summarize neuropathological features described in postmortem human COVID‐19 brains (*n* = 352). Furthermore, analysis of biofluids and neuroimaging from PASC patients underline long‐term changes in the proteome and CNS response following the infection. Postmortem brain studies from severe COVID‐19 patients highlight disruption of the fluid‐brain barriers and vascular dysregulation defined by endothelial inflammation and disruption, hemorrhages, and hypoxic–ischemic damage. Neuroinflammation, including astrogliosis, microglia nodules and infiltration of adaptive immune cells, has been reported in the olfactory bulb, medulla oblongata, midbrain and cerebellum. Neuronal damage was demonstrated in the hippocampus, midbrain and cerebellum in severe COVID‐19 and protein aggregation was observed in the midbrain and entorhinal cortex. Neuropathological burden and elevated blood and/or cerebrospinal fluid (CSF) levels of proinflammatory cytokines (e.g. IL‐6) and neuro‐axonal proteins (e.g. NfL) correlated with severity of anosmia, memory deficits, and cerebellar ataxia. Elderly patients and/or patients with underlying neurological diseases were more susceptible and had worsened symptoms. Potential disease mechanisms underlying neurological symptoms observed in severe COVID‐19 are vascular and fluid‐brain barrier abnormalities, chronic neuroinflammation, persistent axonal damage and protein aggregation. In PASC patients, an altered biofluid proteome with increased neuronal proteins and pro‐inflammatory cytokines was observed. The pathological burden in affected brain regions may contribute to manifestations such as anosmia, memory deficits, and cerebellar ataxia.

## Graphical abstract

Severe COVID‐19 is associated with vascular dysregulation and chronic neuroinflammation, leading to axonal injury and neurodegeneration. In long COVID or PASC, persistent alterations in neuroimaging and biofluid biomarkers reflect ongoing neuronal damage and neuroinflammation, contributing to long‐term neurological symptoms including fatigue, cognitive dysfunction, and mood disorders.

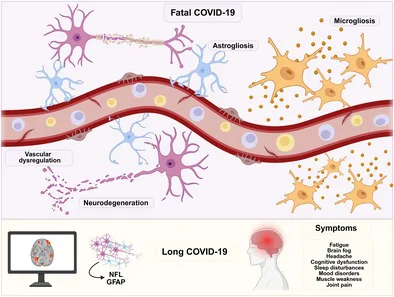

## 1 INTRODUCTION

Infection with SARS‐CoV‐2 may lead to a systemic disease known as coronavirus 2019 (COVID‐19). SARS‐CoV‐2 was identified as a virus transmittable by airborne particles and droplets and demonstrated an accelerated worldwide spread [1, 2]. To date, there have been over 800 million confirmed cases and 7 million deaths globally caused by an infection with SARS‐CoV‐2 reported by the World Health Organization [3]. Although severe COVID‐19 cases are now relatively rare, millions of people continue to experience persistent symptoms, and the long‐term neurological consequences remain largely unknown. In fact, it has been reported that at least 30% of infected individuals display sustained symptoms for weeks or months after recovery, giving rise to the term long COVID, also known as PASC (Table 1) [6, 9, 10]. For consistency, throughout this review we will refer to this condition as “PASC”.

**TABLE 1:** Classification of COVID‐19 terms.

Definition	Description	References
Severe	Acute respiratory distress syndrome (ARDS) Severe pneumonia Hypoxia Hospitalization (ICU, mechanical ventilation)	Flacco et al. [4] and Schurink et al. [5]
Fatal	Acute organ failure Progressive respiratory failure Comorbidities	Flacco et al. [4] and Schurink et al. [5]
PASC/long COVID	Symptoms persist >3 months Fatigue, ‘Brain fog’, headache, cognitive dysfunction Sleep disturbances, mood disorders Muscle weakness, joint pain	Davis et al. [6], Masserini et al. [7] and Nicotra et al. [8]

Although asymptomatic SARS‐CoV‐2 infection can occur (~40%–60% of cases) [11, 12], COVID‐19 patients typically present with symptoms as fever, cough, myalgia, and fatigue [13, 14]. In more severe cases, similar to those observed in severe acute respiratory syndrome (SARS, caused by SARS‐CoV‐1) and middle east respiratory syndrome (MERS, caused by MERS‐CoV), COVID‐19 patients may also experience dyspnea and pneumonia possibly requiring assisted ventilation in an intensive care unit (Table 1) [13, 14, 15, 16]. Common symptoms of PASC include fatigue, respiratory symptoms (e.g., dyspnea, cough and throat pain) and musculoskeletal pain (e.g., myalgias and arthralgias) [7, 17, 18, 19]. In addition, neurological symptoms are common, including brain fog, headache, memory loss, sleep problems, dizziness and balance issues, anosmia, depression and autonomic dysfunction [8, 10, 20, 21, 22, 23, 24, 25]. Although the SARS‐CoV‐2 variant and duration of infection play a role in the severity and/or prevalence of observed symptoms, this is not completely elucidated and often not reported [6]. An infection with the earlier variants, alpha and delta, potentially caused a higher risk of developing PASC symptoms than the later omicron variants; this could be explained by the viral strain, but also by protection caused by vaccination that was still unavailable at the beginning of the pandemics [26, 27, 28, 29]. PASC significantly impacts a patient's quality of life [6, 8, 30, 31], underlying the urgent need for a deeper understanding of the disease mechanisms associated with the neurological symptoms caused by the infection.

Historically, pandemics taught us that the long‐term neurological effects of viral infections can manifest decades after exposure to the virus [32]. The greatest example is the Spanish flu pandemic, which occurred between 1918 and 1920, after which there was an increase in the incidence of parkinsonism cases. Those born during the pandemic were two to three times more likely to develop parkinsonism than those born either before 1888 or after 1924 [33]. Individuals developed postencephalitic parkinsonism (PEP), which is common following encephalitis lethargica disease [34]. Some of these PEP patients showed neurofibrillary tangles in various brain regions such as the substantia nigra, locus coeruleus, pons, hypothalamus, and hippocampus, similar to what is seen in Alzheimer's disease (AD) [35]. Beyond historical observations, strong associations between viral infections and neurodegenerative diseases have been demonstrated in large cohort studies. Levine et al. identified 22 replicated associations between viral infections and neurodegenerative diseases, with the strongest being between viral encephalitis and AD [36]. Taking into consideration what has happened in the past and the new knowledge from recent research on COVID‐19, the relationship between SARS‐CoV‐2 and neurodegenerative diseases is receiving increasing attention from the scientific community. Patients with COVID‐19 and those with neurodegenerative diseases share several similarities, including a pro‐inflammatory biofluid profile, dysfunction of the microvasculature, and sustained neuroinflammation [6, 37]. Whether SARS‐CoV‐2 directly causes or accelerates neuropathology is not yet fully understood [6, 37]. Studying postmortem brain tissue and biofluids collected from COVID‐19 patients is crucial for understanding the intricate relationship between the infection, the brain, and the increased risk for progressive neurodegenerative diseases.

Different terminologies have been proposed in the literature to describe “severe” or “fatal” COVID‐19. Severe COVID‐19 patients were described as critically ill and hospitalized with acute respiratory syndrome, severe pneumonia, and hypoxia; while fatal COVID‐19 is characterized by acute organ and progressive respiratory failure and patients deceased caused by the infection. Both terminologies are used in the literature, and they will be described in this review (for classification see Table 1). On the other hand, when symptoms persist for months after infection, the term PASC is used (for classification see Table 1). The aim of this narrative review is to summarize the main neuropathological features of COVID‐19 (*n* = 352 brains) observed in severe and fatal COVID‐19 patients. This review includes studies published between 2020 and 2025 that describe neuropathological features in human postmortem COVID‐19 brain tissue. As there is no postmortem PASC tissue, relevant neuroimaging and biofluid studies were included in this review. Using key words in PubMed, as “human neuropathology”, “COVID‐19”, “vascular”, “inflammation”, “neurodegeneration”, “severe” and “PASC” we found 196 studies which address the key neuropathological features following infection (for an overview see Table 2).

**TABLE 2:** Overview of neuropathological features in severe and fatal postmortem human brain tissue as well as PASC patient biofluids.

Topic	Neuropathological features in COVID‐19	Brain regions and body fluids	References
Vascular manifestations	Hemorrhagic stroke/microbleeds	Subcortical white matter, olfactory bulb, cerebral arteries, hippocampus, thalamus, cerebellum, grey matter, frontal lobe, brainstem, choroid plexus	von Weyhern et al. [38], Gelpi et al. [39], Thakur et al. [40], Colombo et al. [41], Al‐Dalahmah et al. [42], Kantonen et al. [43], Wierzba‐Bobrowicz et al. [44] and Reichard et al. [45]
	Ischemic stroke/ global ischemia/hypoxic injury		Matschke et al. [46], Rosu et al. [47], Emmi et al. [48]. von Weyhern et al. [38], Gelpi et al. [39], Thakur et al. [40], Lee et al. [49], Schurink et al. [5] and Reichard et al. [45]
NVU inflammation and BBB integrity	Serum protein leakage (Fibrinogen, IgM)		Lee et al. [49], Bocci et al. [50], and Song et al. [51]
	TJ1(−), PECAM(↑), PDGFRβ(↓)		Lee et al. [49], Rosu et al. [47], Zhang et al. [52], Song et al. [51] and Bocci et al. [50]
	IFN response, pro‐inflammatory cytokines, pro‐inflammatory astrocytes and endothelial cells		Chen et al. [53], Krasemann et al. [54], Zhang et al. [52], Suzzi et al. [55], Yang et al. [56], Beiersdorfer [57], Rosu et al. [47] and Dhariwal et al. [58]
Signs of neuroinflammation	Pro‐inflammatory microglia Markers: IBA1(↑), CD68(+), HLA‐DR(+), P2RY12(↓), IFN	Olfactory bulb, medulla oblongata, cerebellum, frontal cortex, subcortical white matter, vagus nerve	Matschke et al. [46], Zhang et al. [52], Emmi et al. [48], Gelpi et al. [39], Poloni et al. [1], Thakur et al. [40], Lee et al. [49], Colombo et al. [41], Schurink et al. [5], Fekete et al. [59], Al‐Dalahmah et al. [42], Deigendesch et al. [60], Lengacher et al. [61], Radke et al. [62] and Woo et al. [63]
	Pro‐inflammatory astrocytes Markers: GFAP(↑), AQP4(↓), CHI3L1(↑)		Matschke et al. [46], Zhang et al. [52], Emmi et al. [48], Poloni et al. [64], Lee et al. [49], Colombo et al. [41], Schurink et al. [5], Yang et al. [56], Plantone et al. [65], Chen et al. [53] and Al‐Dalahmah et al. [42]
	Immune cell infiltration Markers: CD3(+), CD4(+), CD8(+), CD20(+), CD68(+), CD163(+)	Blood plasma and serum, CSF	Matschke et al. [46], Zhang et al. [52], Rosu et al. [47], von Weyhern et al. [38], Gelpi et al. [39], Poloni et al. [64], Thakur et al. [40], Lee et al. [49], Schurink et al. [5], Fekete et al. [59], Schwabenland et al. [66], Soung et al. [67], Schwabenland et al. [68] and Mohme et al. [69]
	Increased pro‐inflammatory cytokines Markers: IL‐1β(+), IL‐6(+), IL‐8(+), IL‐12(+), IL‐18(+) and TNFα(+)	Blood plasma and serum, CSF	Zhang et al. [52], Gomes et al. [70], Huang et al. [13], Queiroz et al. [71], Reinhold et al. [72], Xu et al. [73], Greene et al. [74], Nuber‐Champier et al. [75] and Fernandez‐Castaneda et al. [76]
Neuronal damage	Signs of cell death, neuronal loss axonal and myelin degeneration Markers: NfL(+), GFAP(↑), TH(↓)	Hippocampus, substantia nigra, striatum, caudate nucleus, grey matter, cerebellum, DMN, medulla oblongata, basal nuclei, cranial nerve V, nucleus tractus solitarii, dorsal raphe nuclei, fasciculus longitudinalis medialis	Zhang et al. [52], von Weyhern et al. [38], Gelpi et al. [39], Poloni et al. [64], Colombo et al. [41], Schurink et al. [5], Thakur et al. [40], Yang et al. [77], Lennol et al. [78], Hay et al. [79], Plantone et al. [65], Chen et al. [53], Gutman et al. [80], Smeele et al. [81], Al‐Dalahmah et al. [42], Kantonen et al. [43], Radke et al. [62] and Seo et al. [82]
Protein aggregation	Aβ(+), p‐tau (+), pSer129‐syn(+), TDP43(+)	Olfactory cortex, substantia nigra, temporal lobe, frontal cortex Blood serum	Priemer et al. [83], Zhang et al. [84], Gelpi et al. [39], Thakur et al. [40], Agrawal et al. [85], Duff et al. [86] and Lengacher et al. [61]

*Note*: (↑) increased expression, (↓) decreased expression, (+) positive, (−) negative cells/neurons/proteins.

## 2 POTENTIAL ROUTES FOR SARS‐CoV‐2 ENTRY INTO THE CNS

The SARS‐CoV‐2 virus can reach numerous organs such as the lungs, heart, small intestines, colon and thyroid gland by entering cells through binding to angiotensin‐converting enzyme 2 (ACE2) receptors, transmembrane serine protease 2 (TMPRSS2) or priming of the spike (S) protein and basagin (CD147) [87, 88]. These receptors are mainly expressed on epithelial cells, yet mesenchymal and endothelial cells also demonstrate ACE2 and TMPRSS2 expression [89, 90]. The S protein is a structural surface protein that binds to the ACE2 receptor initiating cell entry of the virus [91]. Investigations of how the virus can potentially enter the CNS have given rise to numerous theories on neuroinvasive routes, including the hematogenous route, extracellular vesicles (EVs), the gut‐brain axis, and the olfactory route.

### 2.1 Limited evidence for direct SARS‐CoV‐2 neuroinvasion

However, SARS‐CoV‐2 RNA was not found in the CSF of COVID‐19 positive individuals [92, 93, 94, 95, 96]. In addition, only very little evidence for the presence of SARS‐CoV‐2 RNA or proteins has been produced in olfactory regions, brainstem, and cerebellum, namely those regions that show neuropathological features in fatal COVID‐19 patients [48, 97]. Notably, several studies have reported no detectable virus in the brain at all [5, 49, 66, 98]. Reports of viral replication in the CNS are very limited, suggesting that the observed neuropathological manifestations are more likely caused by indirect mechanisms rather than direct viral presence.

### 2.2 Hematogenous route

The SARS‐CoV‐2 virus can make its way into the blood circulation and, although there is no compelling evidence that the virus can cross the blood–brain barrier (BBB), the S protein has been detected in endothelial cells of the cerebellum and leptomeninges [97]. Another study by Rong et al., in severe COVID‐19 patients, found SARS‐CoV‐2 RNA (6/12 patients) and S protein (9/12 patients) in the dura, arachnoid, and pia mater [99]. Moreover, epithelial cells and cells within the stroma of the choroid plexus (CP) express ACE2 receptors. Detection of the S protein in the CP of COVID‐19 patients also implicates the blood‐cerebrospinal fluid barrier (BCSFB) as a potential viral entry route [100, 101, 102].

### 2.3 Extracellular vesicles

EVs are secreted nanovesicles enveloped by a lipid bilayer that are crucial mediators of intercellular communication [41, 103]. They carry cell‐signaling molecules (e.g., lipids, proteins, micro RNA), which can modulate the function of target cells once delivered [104, 105]. Certain studies suggested EV transport of SARS‐CoV‐2, as some EVs were SARS‐CoV‐2 RNA‐positive [106]. Troyer et al., observed in vitro that EVs transporting the S protein also could contain serum‐derived neutralizing antibodies [107, 108]. Additionally, EVs isolated from blood plasma of severe COVID‐19 patients showed luminal protein signatures associated with inflammation, coagulation, immune regulation and tissue remodeling which also correlated with disease severity (e.g., the World Health Organization clinical progression score) and hospitalization [109, 110]. These EVs displayed higher levels of inflammatory markers as CD41, CD4, CD8 and CD14, and contained higher levels of interleukin‐6 (IL‐6) [111]. EVs from the plasma of COVID‐19 patients demonstrated enhanced levels of SARS‐CoV‐2 and mitochondrial proteins, especially in PASC patients [112]. Additionally, in EVs isolated from the serum of PASC patients (12 out of 14), SARS‐CoV‐2 associated proteins were found, which play a role in viral RNA transcription and replication. These proteins were absent in serum EVs from pre‐pandemic subjects [113].

### 2.4 Gut–brain axis

ACE2 expression as well as the S and nucleocapsid (N) protein, which is a structural protein playing a role in viral RNA assembly, have been observed in the hypoglossal nerve and dorsal motor nucleus (DMN) of the vagus nerve after SARS‐CoV‐2 infection [46, 48]. Furthermore, viral RNA together with infiltration of immune cells was detected in the vagus nerve of fatal COVID‐19 patients [63]. The vagus nerve senses the microbiota metabolites in the gut and transfers this information back to the CNS [114]. The intestinal wall shows a high expression of ACE2, making it susceptible to viral infiltration. Interestingly, COVID‐19 is commonly characterized by gastrointestinal disturbances, especially in children, whose level of gastrointestinal ACE2 expression is higher [115, 116, 117]. This often leads to intestinal dysbiosis in COVID‐19 patients with an increase of pro‐inflammatory and potentially neurotoxic gut microbiota [118, 119]. Emerging evidence suggests that microbiome dysbiosis, inflammatory signaling, and immune cell dysregulation induced by viral infections may contribute to neuroinflammation and predispose the brain to neurodegeneration [120, 121].

### 2.5 Trigeminal nerve and olfactory system

SARS‐CoV‐2 can potentially enter via the olfactory mucosa and reach the olfactory epithelium and connected regions in the CNS [97, 122]. Sustentacular cells in the human olfactory mucosa express ACE2 receptors [122, 123]. Viral RNA copies were detected in the olfactory mucosa, and to a much lesser extent in the olfactory tubercle [97, 124]. Additionally, viral RNA copies were found in branches of the trigeminal nerve and its ganglion (3 out of 33 patients) of severe COVID‐19 patients [97, 124]. Viral RNA and the S protein were also detected in connecting brain regions (olfactory bulb [3 of 33 patients], medulla oblongata [6 of 33 patients] and cerebellum [3 of 33 patients]), which are implicated with anosmia and respiratory symptoms [97, 124]. Another study found neurons positive for the S or N protein in the brainstem and specifically in the dorsal medulla oblongata and substantia nigra (10 of 24 patients) [48]. SARS‐CoV‐2 S protein colocalized with neuronal cells in the olfactory mucosa of the same severe COVID‐19 patients [97].

## 3 NEUROPATHOLOGICAL FEATURES IN THE POSTMORTEM HUMAN BRAIN FOLLOWING SARS‐CoV‐2 INFECTION

### 3.1 COVID‐19 associated vascular abnormalities

The vasculature of the brain can be significantly affected by a SARS‐CoV‐2 infection. There is an increased risk of cerebrovascular disease, which seems to correlate with age and hospitalization of the patients [37]. Owens et al., described that cerebrovascular abnormalities including cerebral small vessel disease, subarachnoid hemorrhage, hypoxic–ischemic damage, and thromboembolic events, were the most prevalent vascular pathologies in the brains of COVID‐19 patients [125]. Structural changes such as tissue injury, edema and microhemorrhages were observed in subcortical white matter and the olfactory bulb using computer tomography (CT) or magnetic resonance imaging (MRI) in severe COVID‐19 patients [126, 127, 128]. These abnormalities are believed to be caused by a hypercoagulable state, cardiomyopathy, inflammation and endothelial damage [39, 101, 129]. All processes were linked to the binding of the virus to ACE2 receptor or the presence of the S protein in endothelial cells [130, 131]. Matschke et al., observed no evidence of cerebral bleeding or small vessel thromboses. However, 6 of 43 patients showed fresh ischemic infarcts: 3 in the territory of the posterior cerebral artery, 2 of the anterior cerebral artery, and one of the middle cerebral artery, which were more likely to be associated with thromboembolic events [46]. Reichard et al. and Kantonen et al., observed perivascular acute disseminated encephalomyelitis‐like vein‐centered pathologies and hemorrhagic white matter lesions present throughout the cerebral hemispheres [43, 45]. Notably, hemorrhagic white matter lesions can also be linked to axonal injury and macrophage infiltration [45]. Microvessels were also found to be affected demonstrating triggered classical complement cascade caused by immunoglobulin complexes, endothelial cell activation by cytokines, and autoantibodies directly bound to vascular antigens [49]. Diffuse hypoxic–ischemic damage was observed in several patients with severe COVID‐19, especially in hippocampal, thalamic, cerebellar and dentate neurons [39]. Vascular hypoxia was seen in fatal patients demonstrating middle and posterior arterial pathology, including neocortical infarcts, small hemorrhages, white matter lesions and vasculitis [132, 133, 134]. Agrawal et al., showed that 11 of 22 brains had recent acute or subacute ischemic lesions in the parietal, temporal and frontal regions, and in 7 brains intravascular microthrombi were also observed. Interestingly, severe cases resembled acute hemorrhagic leukoencephalopathy and others showed vasculitis‐like changes including fibrinoid necrosis and inflammation in multiple vessel walls [43, 85]. Rhodes et al., observed paravascular and petechial hemorrhages and acute neutrophilic vasculitis in 25 out of 36 fatal cases [135]. Two studies reported subarachnoid hemorrhages in the cerebral hemispheres, pons, medulla and cerebellum in fatal COVID‐19 tissue [42, 44]. In a recent study, cerebral microvascular dysfunction was measured by diffusion MRI 9 months post‐infection and was more prominent in severe COVID‐19 compared to mild patients [136]. Observations in the normal appearing white matter suggested increased cerebral tissue hypoxia during acute infection associated with persistent subjective cognitive deficits [136]. These findings demonstrate that COVID‐19 can have a significant impact on the brain's vascular system contributing to the influx of inflammatory cytokines and immune cells.

### 3.2 The neurovascular unit dysregulation in COVID‐19 patients

The neurovascular unit (NVU) is a multicellular complex consisting of endothelial cells, pericytes, astrocytes, microglia and neurons. It is the interface between blood and brain parenchyma and regulates the exchange of circulating molecules (e.g., cytokines and glucose), oxygen, immune cells and metabolic waste removal. The BBB is an essential part of the NVU formed by endothelial cells which are sealed together by tight junction (TJ) proteins [137, 138, 139]. These BBB properties of endothelial cells are sustained by astrocytic endfeet that cover most of the BBB surface, also regulating vessel dilation and constriction [140, 141, 142]. SARS‐CoV‐2 is known to provoke the so‐called ‘cytokine storm’ caused by systemic inflammation and characterized by massive changes in pro‐ and anti‐inflammatory cytokines [143, 144]. IL‐6, tumor necrosis factor alpha (TNFα), monocyte chemotactic protein‐1 (MCP1) and interferon‐gamma (IFNγ) are increased in severe COVID‐19 biofluids [70, 71, 72, 73, 74]; these levels correlated with disease severity and memory performance months after infection [74, 75]. The ‘cytokine storm’ can have detrimental effects on the NVU causing vascular injury and increased permeability [145, 146] (Figure 1). The NVU in the cortical gray matter from severe COVID‐19 patients showed increased interferon induced transmembrane protein (IFITM) expression, which is an anti‐viral response caused by activation of the type I interferon (IFN) signaling pathway [53, 54]. BBB disruption was correlated with cytokine IL‐6 and IL‐8 levels in plasma and CSF from severe COVID‐19 patients [147, 148, 149, 150]. In blood collected from PASC patients, Bonetto et al., observed higher levels of matrix‐metalloproteinase‐9 (MMP‐9), also pointing to BBB damage [151]. Endothelial cells in the NVU of COVID‐19 patients are dysregulated caused by loss of TJ proteins (e.g., TJ1) and increased activation markers (e.g., platelet endothelial cell adhesion molecule (PECAM‐1)) [47, 49, 52] (Figure 1). Additionally, intracellular adhesion molecule 1 (ICAM1) was enriched in blood vessels, and leukocytes containing MMP‐9 and aquaporin‐4 (AQP4) were decreased in astrocytic endfeet, all pointing to inflammation‐induced BBB dysregulation [59]. Various pathological changes occur at the BBB, such as swelling of endothelial cells and retraction of astrocytic endfeet surrounding blood vessels in the frontal lobe, brainstem and hippocampus [47, 52]. In turn, this can lead to enlarged perivascular spaces and edema [52, 132]. Perivascular astrocytes have shown depolarized AQP4, elevated glial fibrillary acidic protein (GFAP) expression and enlarged cell bodies with thickened processes [47, 57] (Figure 1). Notably, an increase of fibrinogen and immunoglobulin M (IgM) in the parenchyma of COVID‐19 patients showed a leaky BBB [49, 51] (Figure 1). In PASC patients, lower soluble levels of the pericyte marker platelet‐derived growth factor receptor beta (PDGFRβ) were observed in the CSF suggesting loss of pericytes [50, 152]. Zhang et al., found a downregulation of extracellular matrix (ECM) components such as laminins and collagens [52]. Additionally, collagen disruptions co‐occurred with TJ loss [47, 49] in COVID‐19 brain regions (e.g., cortex, hippocampus, brainstem) suggesting vascular basement membrane dysregulation. However, Zhang et al., showed that following a SARS‐CoV‐2 infection, disruption of the basement membrane of endothelial cells was also observed without alterations of TJ structure and expression [153]. A dysfunction of mitochondria leading to ECM dysregulation, endothelial cell activation, pro‐inflammatory cytokine release and oxidative stress in the BBB was associated with COVID‐19 induced neurological symptoms [58]. The inflammation, dysregulation and increased permeability of the BBB during COVID‐19 can conceivably contribute to neuroinflammation [125, 145, 146].

**FIGURE 1:**
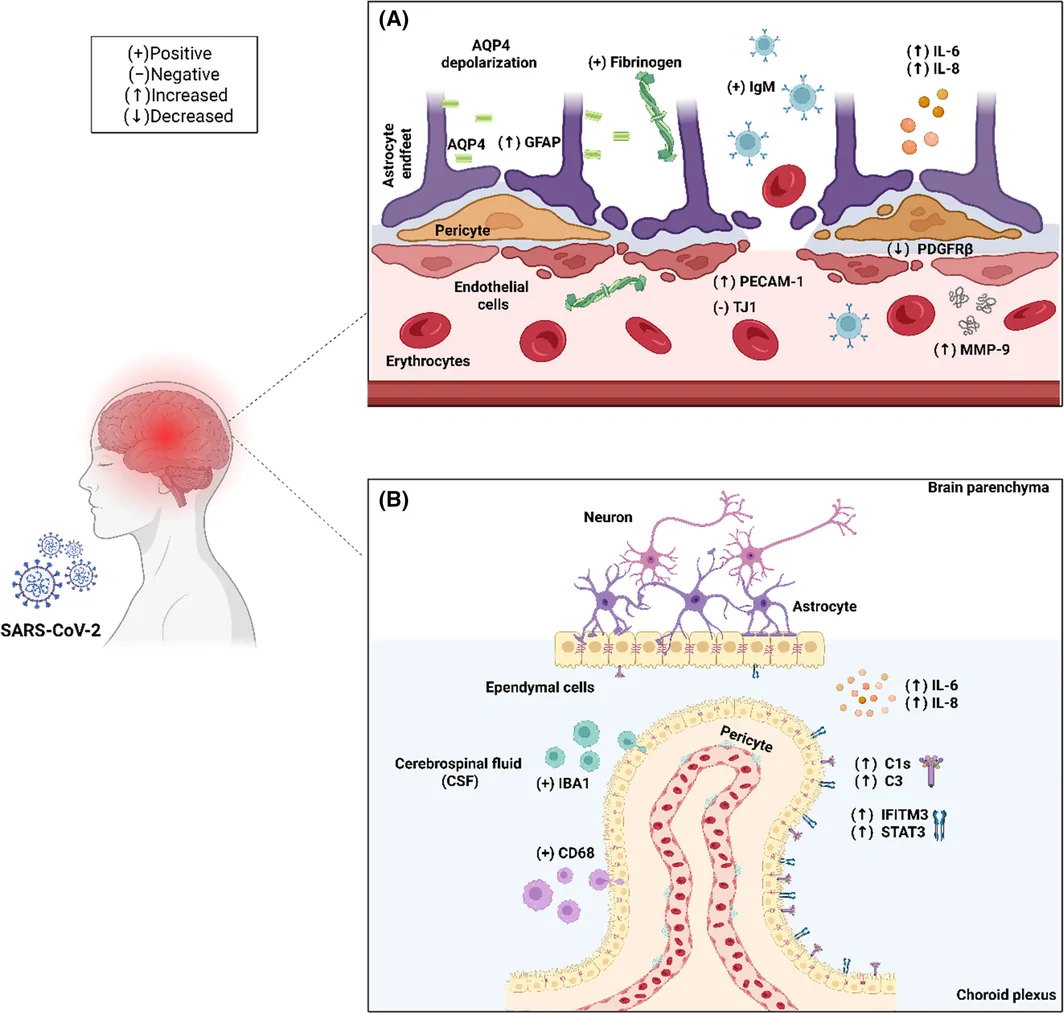
Damage to the blood–brain barrier (BBB) and blood‐cerebrospinal fluid barrier (BCSFB) in COVID‐19 patients. A dysfunctional BBB in COVID‐19 patients is characterized by endothelial cell dysregulation caused by decrease of TJs, changes in astrocyte morphology, degradation by MMP‐9 and activation with increased PECAM‐1 expression. Loss of pericytes resulted in a decreased level of PDGFRβ in the CSF of COVID‐19 patients. Because the increased barrier permeability, there is leakage of blood factors (e.g., fibrinogen and IgM) in the parenchyma. In the BCSFB an increased expression of IFN and complement genes, including IFITM3 and C3, is seen implicating a more inflammatory state. Additionally, activated macrophages and pro‐inflammatory cytokines (e.g., IL‐6 and IL‐8) were observed in the CSF of COVID‐19 patients. (+) positive, (−) negative cells/neurons/proteins, (↑) increased protein expression, (↓) decreased protein expression. Figure was made using BioRender.

### 3.3 Inflammation of the blood‐cerebrospinal fluid barrier in COVID‐19

The BCSFB is made up of fenestrated capillaries of the CP stroma which are separated from the CSF by an epithelial cell layer [102, 154]. Whether the virus enters the CNS via the BCSFB is uncertain and results so far have been inconsistent [56, 92]. CP enlargement, visualized using 3 Tesla MRI, in severe COVID‐19 patients was associated with cognitive dysfunction, reduced grey matter volume, white matter alterations as well as changes in functional connectivity in frontal and subcortical regions, suggesting inflammation and barrier dysfunction [155, 156]. Inflammatory genes of the IFN (e.g., IFITM3) and complement (e.g., C1s and C3) pathways were upregulated in the epithelial and ependymal cells of the CP of severe COVID‐19 patients, even without detecting any viral RNA [55, 56] (Figure 1). Activated ionized calcium‐binding adaptor molecule 1 (IBA1)^+^ or CD68^+^ macrophages were found in the CP [56, 100] of severe postmortem COVID‐19 patients. An increase in the cell‐to‐cell inflammatory pathway communication (e.g., by inflammatory chemokines) between CP cells and cells in the brain parenchyma (e.g., glial cells and neurons) was also observed [56] (Figure 1). The damage of the CP and upregulation of inflammatory pathways can together lead to leakage of immune cells and cytokines into the CSF with subsequent activation of glial cells in the brain parenchyma [102, 157].

### 3.4 Neuroinflammation after SARS‐CoV‐2 infection

Neuroinflammation is characterized by an innate immune response including microgliosis and astrogliosis together with infiltration of adaptive immune cells from the periphery [157]. Several postmortem studies have provided evidence of persistent neuroinflammation in the DMN of the vagus nerve, olfactory bulb, midbrain, brainstem, and cerebellum [5, 46, 49, 66, 158] in patients with severe COVID‐19 (Figure 2). In the olfactory mucosa of COVID‐19 patients CD45^+^ leukocytes, CD4^+^ and CD8^+^ T cells, CD68^+^ macrophages and CD20^+^ B cells were observed infiltrating the tissue [97, 124, 159, 160]. Although there is little if any evidence of viral presence in the olfactory bulb parenchyma [161, 162], infiltrating T cells (CD3^+^ and/or CD8^+^), activated microglia with amoeboid morphology (human leukocyte antigen‐DR isotype (HLA‐DR)^+^) and astrogliosis (GFAP^+^) were found in severe patients [5, 59, 60, 61, 66]. The medulla oblongata, brainstem and cerebellum from fatal patients demonstrated prominent gliosis by means of immunohistochemistry (IHC) with increased GFAP^+^, IBA1^+^, HLA‐DR^+^ and CD68^+^ cells [5, 42, 46, 59, 60, 64, 67, 150]. Increased CD68^+^ cells, amoeboid microglia and reactive astrocytes were also observed in the frontal cortex and subcortical white matter of severe COVID‐19 patients [56, 57, 76, 150]. Fekete et al. and Yang et al., showed increased microgliosis and decreased expression of the microglia homeostasis marker purinergic receptor P2Y12 (P2RY12) in the medulla of severe COVID‐19 patients. In line with this findings, transcriptomic changes in the brainstem of acute patients showed increased IFN responses upregulated by microglia, macrophages and endothelial cells, including decreased microglial P2RY12 expression [62]. Moreover, Yang et al., observed transcriptomic changes in astrocytes in the frontal cortex, which expressed IFITM3, GFAP and chitinase 3‐like 1 (CHI3L1) characterizing an inflamed and neurotoxic phenotype [56]. CD3^+^ and cytotoxic CD8^+^ T cells were also present in the medulla oblongata, brainstem, and cerebellum of severe COVID‐19 patients with perivascular cuffing of T cells and formation of microglia‐T cell nodules (CD68^+^HLA‐DR^+^CD8^+^) [5, 40, 66, 68, 69]. In addition, increased perivascular CD163^+^ macrophages and infiltrated CD20^+^ B cells were present in the medulla of severe COVID‐19 patients [59, 66]. Immunophenotyping and sequencing studies have reported immunological changes in the blood of PASC patients mainly consisting of T‐cell dysfunctions [163, 164]. Six to 8 months post‐infection, increased CD4^+^ T cells and exhausted SARS‐CoV‐2‐associated CD8^+^ T cells were detected [164, 165]. Increased senescent CD8^+^ T_EMRA_‐lymphocytes together with increased inflammatory gene expression in monocytes and pro‐inflammatory cytokines were demonstrated (e.g., IFNγ, IL6 and IL‐17a) in the blood of PASC patients, 3–6 months post‐discharge from the hospital [163, 166]. An exacerbated neuroinflammatory response involving microglia/macrophages and astrocytes was visualized using positron emission tomography (PET) by increased translocator protein 18 kDa (TSPO) ligand [^18^F]DPA‐714 in PASC patients [167]. Patients with neurological complaints demonstrated this increased inflammatory response in the brain, which was diminished in cognitively unimpaired cases. This inflammatory response was widespread throughout the brain but did not show prevalence in a specific brain region [167]. [^11^C]PBR28, a different TSPO ligand, was increased in PASC individuals in various brain regions as the anterior cingulate cortex, thalamus and basal ganglia. This visualized neuroinflammation using PET positively correlated with peripheral proteins associated with vascular dysfunction, suggesting a link between the processes during the disease [168]. In another PET study, the astrogliosis marker [^11^C]SL25.1188 was increased in the prefrontal cortex, hippocampus and striatum of PASC patients and negatively correlated with depressive symptoms [169]. These persistent neuroinflammatory manifestations in the brain could potentially contribute to neuronal damage [5, 49, 170].

**FIGURE 2:**
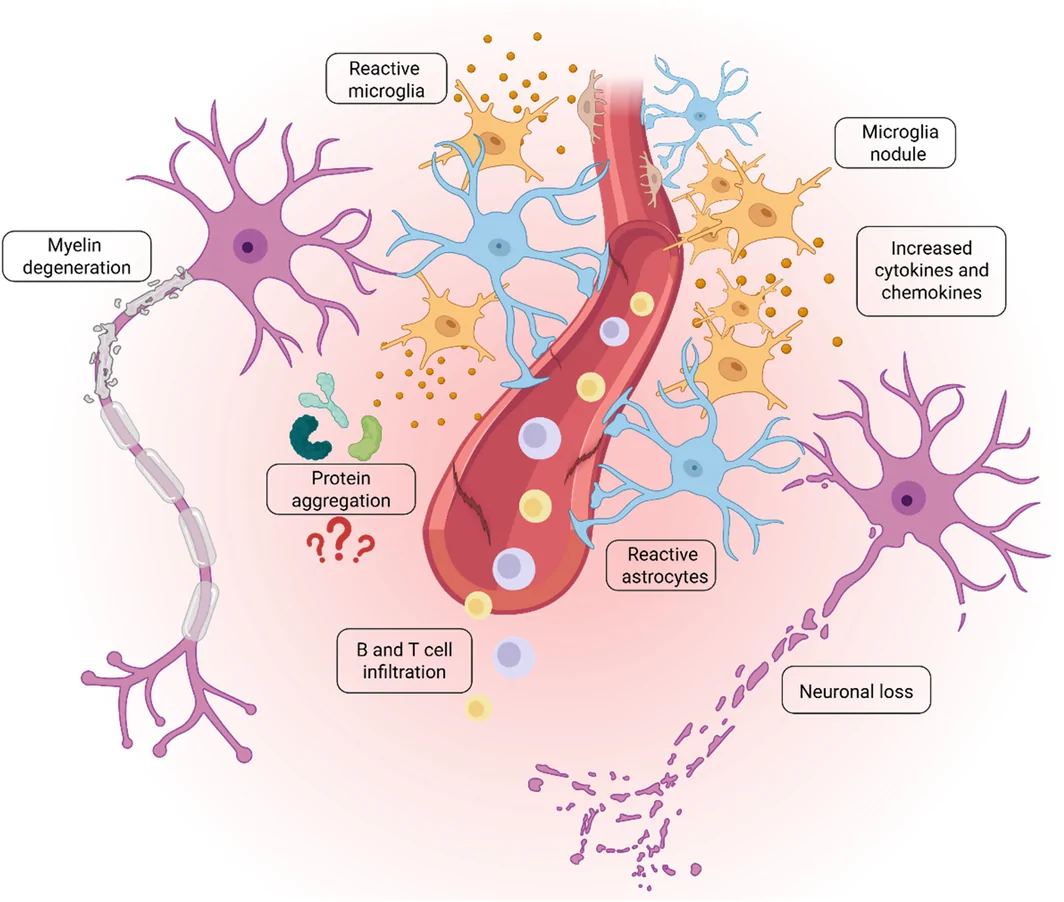
Schematic representation of neuropathological findings in severe COVID‐19. Postmortem brain tissue from COVID‐19 patients demonstrated extensive neuroinflammation with microgliosis, astrogliosis, and infiltration of peripheral immune cells and inflammatory molecules. This neuroinflammatory response may contribute to neuronal damage, myelin degeneration, and potentially protein aggregation. Figure was made using BioRender.

### 3.5 Neuronal damage

There is growing evidence that SARS‐CoV‐2 can trigger and/or potentially accelerate neurodegenerative processes in individuals with underlying vulnerabilities, ‘at risk’ or in prodromal stage of the disease. An example is given by patients with Creutzfeldt‐Jakob disease who have shown a rapid disease progression following a SARS‐CoV‐2 infection [171]. Additionally, as demonstrated by Yu et al., individuals with neurodegenerative diseases such as AD had a higher mortality risk, while Parkinson's disease (PD) also showed a strong association with SARS‐CoV‐2 infection but with lower mortality [172]. Numerous studies have reported neuronal damage in individuals with severe COVID‐19, highlighting potential effects of the infection on the CNS [52, 79] (Figure 2). However, most of these patients were elderly and had pre‐existing neurological conditions. Studies have shown elevated levels of neurofilament light chain (NfL), a marker for axonal damage and neuronal degeneration, in plasma [78] and serum of COVID‐19 patients [79] even in the absence of neurological complications. Yet, studies found increased NfL levels within a month in severe and PASC patients, correlating with delirium duration, fatigue, severity markers such as ‘Sequential Organ Failure Assessment’ scores and inflammatory markers (e.g., TNFα and IL‐1β) [80, 81]. However, soluble GFAP and NfL levels were also increased following asymptomatic and mild SARS‐CoV‐2 infection, with even higher serum levels in patients with cognitive impairment, 1 week [65] and even still 1 year [82] after SARS‐CoV‐2 infection. The levels of both proteins decreased after 10 months in the same patients yet still remained above the levels in healthy controls [65]. These results implicate ongoing astroglial and neuronal damage in severe COVID‐19 and PASC, which could explain some of the persisting neurological symptoms [65, 81].

It has been suggested that neuroinflammation may contribute to neuronal injury through oxidative stress [173]. Spatial transcriptomics showed a decreased expression of genes associated with mitochondrial function and synaptic vesicle trafficking, especially in the deep‐layer excitatory cortical neurons in brains of COVID‐19 patients [84]. Additionally, bulk gene expression showed an upregulation of genes related to cell stress and apoptosis, such as caspase‐3 (CASP3) and heat shock protein 70 (HSP70), indicating an increased cellular damage compared to controls with matched pulmonary pathology [84]. In their spatial analysis of the hippocampus of fatal COVID‐19 cases, Bayat et al., observed a disrupted histoarchitecture in the Cornu Ammonis 1 (CA1) and dentate gyrus with an increased polygon area and space per cell indicating neuronal loss [174]. Moreover, elevated apoptosis in pyramidal and granular neurons (via cleaved CASP3 marker) and reduced neurogenesis were present in the dentate gyrus. Shorter dendrites and fewer dendritic spines in CA1 pyramidal neurons were shown as well as reduced neuronal branching suggesting impaired synaptic plasticity, memory and learning [174]. In COVID‐19 associated monocytic encephalitis postmortem brain studies, Zangh et al., discovered damage to neurons and the myelin sheath, which was linked to dysregulation of HLA‐DR, CD163, and other proteins in monocytes. In the grey matter, acute neuronal injury was shown along with the loss of Nissl bodies and the appearance of giant neurons and swollen axons. Additionally, there was a reduction in the number of cerebellar Purkinje and granular cells [52], which was also demonstrated by Colombo et al. [41]. Electron microscopy and Luxol fast blue staining revealed swollen, fragmented and loss of myelin sheaths in the white matter [43, 52, 64]. In severe COVID‐19 patients, neuronal damage in the DMN of the pons and the reticular formation was noted alongside cellular alterations as condensed chromatin, shrunken cell bodies and neuronal chromatolysis [41]; in the medulla oblongata axonal damage and myelin sheath changes were also demonstrated [41, 175]. Shrunken neurons were also observed in an acute case study by Al‐Dalahmah et al., in different brain regions, including the cerebral cortex, midbrain, pons and cerebellum. Moreover, Colombo et al., observed thicker and curved axons with axonal swellings in the basal nuclei and the medulla oblongata [41]. Disruption of the neuropil was characterized by vacuolized neurons visualized with anti‐neurofilament immunolabeling resembling the pre‐tangle granular pattern, as seen in Pick's disease and other dementias [41]. In 6 fatal COVID‐19 brains analyzed, von Weyhern et al., found localized perivascular and interstitial encephalitis, neuronal cell loss, and axonal degeneration in specific regions: the DMN of the vagus nerve, cranial nerve V (trigeminal nerve), nucleus tractus solitarii, dorsal raphe nuclei, and fasciculus longitudinalis medialis [38]. Yang et al., showed dopaminergic degeneration in the substantia nigra of severe COVID‐19 patients; both the density of neuromelanin‐containing and tyrosine hydroxylase (TH)‐immunoreactive dopaminergic neurons declined by ~70% and ~40% respectively, and the percentage area of TH‐immunoreactive threads was decreased by ~50% compared to controls [77]. Moreover, Calculli et al., reported on six patients who developed parkinsonism‐like symptoms following SARS‐CoV‐2 infection, with resting tremor as an initial sign. While MRI revealed no brain changes, dopamine transporter (DAT) scans showed reduced uptake in the putamen in four of these six patients [176]. Finally, transcriptomic analysis of the brainstem showed dysregulation of genes in a cluster of dopaminergic neurons; these proteins are associated with synapse biology, including soluble NSF attachment receptor encoding (SNARE), proteins implicated with synaptic vesicle exocytosis [62]. In addition to dopaminergic dysfunction, serotonergic pathways may also be affected in COVID‐19. Serum from PASC patients has shown a reduced serotonin production and accumulation of the excitotoxin quinolinate compared to controls [177], supporting that the tryptophan metabolism might be disrupted. Severe COVID‐19 postmortem brain studies have revealed the significant buildup of tryptophan degradation products, 3‐hydroxy‐anthranilic acid and quinolinic acid, possibly leading to a neurotoxic environment [178, 179]. These accumulations correlated with the expression of indoleamine 2,3‐dioxygenase, which is activated by the hydrocarbon receptor (AHR) [178]. Interestingly, AHR activation in severe COVID‐19 and PASC cases was associated with cellular stress and programmed death, as evidenced by the presence of autophagy and apoptosis potentially contributing to pathology and symptoms [178, 179]. Wong et al., found that the reduction of peripheral serotonin hinders the activity of the vagus nerve in PASC, which in turn impairs hippocampal responses and memory [180]. Viral infections and IFN‐associated inflammatory responses can affect this kynurenine pathway potentially initiating the increased production of neurotoxic and immunosuppressive metabolites [180, 181]. Finally, neurophysiology in COVID‐19 patients also showed neuronal abnormalities; Yao et al., observed altered wave patterns and changes in brain activity through electroencephalogram (EEG) analysis, resulting in altered perception, motor and cognitive function [182]. Similarly, MRI visualized cortical thinning in the cingulate, parahippocampal and insular cortices, including hippocampus, amygdala and caudate nucleus in COVID‐19 patients with olfactory and cognitive impairment [82, 183, 184]. Furthermore, Wood et al., analyzed 351 severe patients and found increased GFAP and NfL serum levels associated with reduced anterior cingulate cortex volume and global cognitive deficits [185]. In addition, Rua et al., demonstrated brainstem microstructural abnormalities causing this region to be more susceptible in post‐hospitalized COVID‐19 patients, using an ultra‐high field 7 T quantitative mapping MRI [156]. Finally, Douaud et al., observed in 401 acute cases, using imaging data from the UK Biobank, atrophy in limbic regions, increase in CSF volume and decrease in whole brain volume indicating loss of grey matter, especially in the olfactory region [186]. These findings provide valuable insights into the contribution of COVID‐19 to neuronal injury and its association with numerous neurological symptoms.

#### 3.5.1 Protein aggregation

The aggregation of proteins can lead to neuronal loss and damage in different neurodegenerative diseases [187, 188]. In in vitro models, SARS‐CoV‐2 and viral proteins have shown to be able to induce protein aggregation of amyloid fibrils and alpha‐synuclein [189, 190, 191, 192, 193]. Whether SARS‐CoV‐2 can potentially cause or set off a cascade towards protein aggregation is not yet elucidated. It has been suggested that the presence of amyloid‐beta (Aβ) plaques, tau tangles and Lewy bodies in postmortem brains of COVID‐19 patients may be age‐related or prominent in individuals who are at risk of cognitive decline [39, 40, 44, 61, 85]. Thakur et al., observed protein aggregation in a subset of severe patients (18 of 41), some of whom were elderly or had previous neurological conditions [40]. Gelpi et al., found alpha‐synuclein immunoreactive Lewy bodies and Lewy neurites in the brainstem (midbrain, pons and medulla oblongata) and olfactory regions of 3 of 32 severe patients, who had not been clinically diagnosed with PD [39]. Phosphorylated Ser129 (pSer129) alpha‐synuclein was increased in the substantia nigra of severe COVID‐19 patients versus non‐COVID‐19 patients together with a loss of TH‐neurons and ‐threads [77]. Lengacher et al., observed pSer129 pathology in three cases of severe COVID‐19 without history of neurological disease and no correlation to age. Moreover, 10 of 32 patients exhibited a combination of tau‐positive neurofibrillary tangles in the transentorhinal region and limbic system and prominent Aβ pathology in cortical areas, limbic system, basal ganglia, brainstem and cerebellum, with or without cerebral amyloid angiopathy (CAA) [39]. These changes were predominantly observed in older individuals (average age approximately 73 years) [39, 40]. Agrawal et al., examined 20 fatal COVID‐19 cases, 4 of whom showed neuronal neurofibrillary tangles in the medial temporal lobe; of these, only one had a previous clinical diagnosis of dementia. Yet, 7 patients had pre‐existing neurological conditions (e.g., AD, Limbic‐predominant age‐related TDP‐43 encephalopathy, PD and Dementia with Lewy bodies), suggesting that their condition was possibly not related to the SARS‐CoV‐2 infection [85]. Furthermore, Aβ deposition was observed in the form of senile plaques in the frontal cortex in 3 of 9 patients, and CAA in 1 of 9 patients, suggesting that the amyloid accumulation could be part of the brain's response to the infection [84]. Aβ accumulation was also shown by Priemer et al., in both severe COVID‐19 patients and in COVID‐19 negative adults who died of acute respiratory distress syndrome, suggesting that the deposits were a result of hypoxia rather than SARS‐CoV‐2 infection [83]. Finally, Aβ pathology was observed in the brain of a severe COVID‐19 non demented case in the anterior olfactory nucleus [61]. Additionally, in the serum of mild COVID‐19 patients, a decrease was observed in the ratio of Aβ proteins similar to AD [86]. Further investigation is required to determine whether COVID‐19 can be considered a potential risk factor for protein aggregation [33, 37].

## 4 CONCLUSIONS

This narrative review explores the neuropathological features of severe COVID‐19, focusing on the brain regions most vulnerable to damage and the possible underlying disease mechanisms. Key findings from these studies involving large cohorts of severe and fatal COVID‐19 patients (~20–50 individuals) highlight several hallmark features, including subarachnoid hemorrhages, BBB dysregulation, microglia‐T cell nodules and reactive astrocytes [46, 49, 59]. These abnormalities have mostly been found in olfactory bulb, hippocampus, cerebellum, medulla oblongata and pons suggesting a possible correlation with the reported symptoms of anosmia, impaired memory and learning, autonomic dysfunction as well as sleep and mood disturbances [5, 46, 49, 59, 158, 160]. Neuroimaging studies in these patients have further revealed significant microvascular dysfunction, hypoxia and microhemorrhages [127, 136], while a persistent neuroinflammatory response was documented in PASC patients [167]. These changes were predominantly observed in subcortical white matter and olfactory bulb linking to neurological symptoms such as fatigue, cognitive deficits and anosmia [74, 127, 136, 167, 194]. Postmortem brain studies unveiled additional neuropathological features in severe and fatal COVID‐19 entailing hypoxic damage, and structural changes such as reduced neuronal branching and damage to axons and myelin sheaths [39, 41, 44, 174, 175]. Neuronal damage, however, remains less studied, especially with regard to how it contributes to persisting cognitive and functional impairments. Although these key neuropathological features were quite consistent across studies, the possibility that severe COVID‐19 might induce protein aggregation remains a debated aspect, as it has been reported in small cohorts (~1–20 individuals) and interpreted either as an incidental finding (Lewy body pathology) or as an age‐related phenomenon (tau tangles and/or Aβ). In fact, a large number of patients had pre‐existing neurodegenerative diseases making it hard to distinguish whether neuronal damage or protein aggregation was pre‐existing or accelerated by the infection [39, 40, 44, 85]. Findings regarding the presence of the virus in the brain remain controversial; some studies report detectable viral load in the brain parenchyma [48], whereas others find no evidence of viral presence [56, 99]. Detection of the SARS‐CoV‐2 virus via immunohistochemical analysis needs to be critically re‐assessed, especially taking correct control tissue and antibodies against different viral antigens into account [97, 195]. Based on these observations, the preferred route of viral entry and the mechanisms underlying CNS responses are yet unclear and require further investigation. The presence of viral proteins in the meninges, EVs, vagus nerve and olfactory neurons has been investigated, but it remains however a matter of ongoing debate if the virus can directly invade the CNS at these points as most studies report no detectable viral RNA in the CNS. These observations suggest that neuropathological manifestations are more likely driven by indirect mechanisms including vascular damage, inflammation and underlying comorbidities. Mechanisms implicated in neuropathology and CNS responses, such as mitochondrial dysfunction, autophagy and apoptosis, are poorly characterized in severe COVID‐19 and PASC patients; investigating these processes in postmortem brain tissue is challenging and could be validated by in vitro and in vivo studies [196, 197, 198]. Notably, postmortem brain studies often involve small sample sizes (~1–20 cases per study), as only a limited number of COVID‐19 brains were collected by brain banks in a standardized manner during or after the pandemic. With regard to PASC, the condition itself is not fatal and affected people die of other causes; therefore, available brain tissue and pathological data from PASC cases are extremely limited. This makes it challenging to define the neuropathology of PASC syndrome and to gain insights into the long‐term consequences of COVID‐19, including the potential increased risk of neurodegenerative conditions. Moreover, there is still no consensus on definition PASC terminology or its heterogeneous phases, which hampers the systematic collection of world‐wide data on this condition. A systematic analysis of neuropathological data of large cohorts of COVID‐19 cases, from different countries and continents is still missing, but is needed to provide a detailed understanding of the large variability in severity and symptoms post‐infection. Finally, current research lacks quantitative neuropathological data allowing more insight in the selective vulnerability of cell types across brain regions.

To conclude, postmortem human brain studies showed neuropathological impacts of severe COVID‐19; however, key questions remain about the long‐term effects as there is limited availability of standardized extensive postmortem brain samples. Multicentric tissue collection is needed today, in order to be ready to face or even be able to anticipate the consequences of the infection tomorrow.

## AUTHOR CONTRIBUTIONS

JMN, AS, MB, WDJB & JM were involved in the conception and design of the review. JMN & AS wrote the original draft of the manuscript. JMN, AS, MB, WDJB & JM critically revised the manuscript. All authors read and approved the content of the manuscript before final submission.

## FUNDING INFORMATION

This project has received funding from the European research project NEUROCOV, funded by Horizon Europe (EU). The views and opinions expressed, however, are those of the author(s) only and do not necessarily reflect those of the European Union or European Health and Digital Agency (HADEA). Neither the European Union nor the granting authority can be held responsible for them.

## CONFLICT OF INTEREST STATEMENT

The authors declare no conflicts of interest.

## Data Availability

Data sharing not applicable to this article as no datasets were generated or analysed during the current study.
